# The Positive Brain – Resting State Functional Connectivity in Highly Vital and Flourishing Individuals

**DOI:** 10.3389/fnhum.2018.00540

**Published:** 2019-01-14

**Authors:** Florens Goldbeck, Alina Haipt, David Rosenbaum, Tim Rohe, Andreas J. Fallgatter, Martin Hautzinger, Ann-Christine Ehlis

**Affiliations:** ^1^Department of Psychiatry and Psychotherapy, University Hospital of Tübingen, Tübingen, Germany; ^2^LEAD Graduate School and Research Network, University of Tübingen, Tübingen, Germany; ^3^Department of Psychology, University of Tübingen, Tübingen, Germany

**Keywords:** flourishing, subjective vitality, functional near-infrared spectroscopy (fNIRS), network-based statistics (NBS), default mode network (DMN), resting state functional connectivity (RSFC)

## Abstract

The World Health Organization has defined health as “complete physical, mental and social well-being and not merely the absence of disease or infirmity” (World Health Organization, 1948). An increasing number of studies have therefore started to investigate “the good life.” However, the underlying variation in brain activity has rarely been examined. The goal of this study was to assess differences in resting state functional connectivity (RSFC) between regular healthy individuals and healthy individuals with a high occurrence of flourishing and subjective vitality. Together, flourishing, a broad measure of psycho-social functioning and subjective vitality, an organismic marker of subjective well-being comprise the phenomenological opposite of a major depressive disorder. Out of a group of 43 participants, 20 high-flourishing (highFl) and 18 high-vital (highSV) individuals underwent a 7-min resting state period, where cortical activity in posterior brain areas was assessed using functional near-infrared spectroscopy (fNIRS). Network-based statistics (NBS) of FC yielded significantly different FC patterns for the highFl and highSV individuals compared to their healthy comparison group. The networks converged at areas of the posterior default mode network and differed in hub nodes in the left middle temporal/fusiform gyrus (flourishing) and the left primary/secondary somatosensory cortex (subjective vitality). The attained networks are discussed with regard to recent neuroscientific findings for other well-being measures and potential mechanisms of action based on social information processing and body-related self-perception.

## Introduction

We know a lot more about the things that can go wrong in life than about the good life (Seligman and Csikszentmihalyi, 2000). For the field of human neuroscience, despite major contributions over the last years (Burgdorf and Panksepp, 2006; Van Reekum et al., 2007; Heller et al., 2009, 2013; Kringelbach and Berridge, 2009; Berridge and Kringelbach, 2015; Kong et al., 2015a,b,c, 2016; Sato et al., 2015; Greene and Seligman, 2016), this is still true. As in the case of psychological disorders, the good life consists of and is being measured in multiple aspects (Ryan and Deci, 2001; Peterson et al., 2005). Two widely used concepts, whose neurophysiological signatures are still unknown, are the constructs of flourishing (Keyes, 2002; Diener et al., 2010; Seligman, 2012) and subjective vitality (Ryan and Frederick, 1997). The term flourishing (Fl) has been used to describe a broad array of distinct dimensions of positive psycho-social functioning (Keyes, 2002; Fredrickson and Losada, 2005; Diener et al., 2010; Seligman, 2012; VanderWeele, 2017) whereas subjective vitality (SV) was introduced as a narrow construct to measure a person’s perception of energy, available for mental and physical action (Ryan and Deci, 2008). In combination, the two concepts mirror the positive opposites of the main non-somatic criteria present in a major depressive episode (Huppert and So, 2013): Feeling competent and engaged, perceiving life as meaningful and being optimistic, experiencing positive emotions, having satisfying relationships and feeling alive and energetic. A healthy person who scores high on these dimensions compared to a healthy person with low scores shows fewer missed days of work, a lower risk for cardiovascular and chronical physical disease and fewer health limitations in daily life activities with age (Pressman and Cohen, 2005; Keyes, 2007). However, despite these findings concerning health and daily life behavior, the differences in human brain activity underlying different levels of Fl and SV have only scarcely been examined. This paper aims at contributing to fill this gap by looking at the neural correlates of flourishing and SV in the brain at rest. We did so via the comparison of highFl and highSV individuals with a group of healthy but regular-flourishing/regular-vital (regFl/regSV) subjects. Flourishing and SV were measured using validated self-report measures (Ryan and Frederick, 1997; Diener et al., 2010). Median split groups were derived for the purpose of group comparison. Both measures, Fl and SV, contain aspects of the “good life” and will be referred to as concepts belonging to the broader area of well-being measures.

Psychological disorders have been studied extensively from a neuroscientific perspective. Hence, we used associated methods and corresponding theories as a starting point for the design and hypotheses in this project. In depression research, recently much attention has been given to changes in RSFC (Wang et al., 2012; Mulders et al., 2015), changes in the temporal correlations of spontaneous brain activity in spatially remote areas in the resting brain (Friston et al., 1993). Some first studies in the field of well-being research also found significant changes in FC associated with happiness (Luo et al., 2015), eudaimonic and hedonic well-being (Luo et al., 2017). The majority of changes thereby occurred in areas of the DMN (Greicius et al., 2003). The DMN anatomically consists of precuneus, adjacent PCC/Rsp, the MPFC, the IPL/AG and the MTC (Horn et al., 2014) as well as parts of the lateral temporal and lateral frontal cortex (Yeo et al., 2011). It is assumed to play a major role in self-referential thought processes (Buckner et al., 2008; Davey et al., 2016). Hence, these processes, in particular rumination, a reoccurring, rather abstract style of thinking about the past or shortcomings of the self, have been highlighted as a potential mechanism for the aberrant FC patterns within the DMN in depression (Rosenbaum et al., 2017). In their study on happiness Luo et al. (2015) found higher resting state FC in the anterior and posterior DMN correlated with an inclination to ruminate and unhappiness. However, in a more recent study, the authors found increased as well as decreased DMN FC, depending on which measure of well-being was applied (Luo et al., 2017). Matching heterogeneity regarding increased and decreased DMN-activity has also been found in the literature on depression (Wang et al., 2012; Mulders et al., 2015; Rosenbaum et al., 2017). Based on these findings of DMN FC variations at rest, we decided to apply a resting state paradigm and measure cortical FC at temporal/parietal areas of the brain with the help of functional near-infrared spectroscopy (fNIRS). As part of an on-going project to study positive human neuroscience in more naturalistic contexts, we used fNIRS because the method combines relatively high temporal resolution, mobile application, insensitivity to movement artifacts, low costs and easy assessment (Ehlis et al., 2014). NBS were used to detect significant network differences in FC between the groups. As the tendency to ruminate has shown to be relevant for differences in DMN FC, we included a trait and state measure to account for this. Furthermore, to also cover mental activity at the other side of the spectrum, we assessed the feeling of free flowing thoughts (mind-wandering) during the measurement. Mind-wandering in this sense, has been proposed as opposite mental state to rumination (Rosenbaum et al., 2017). To control for general subjective experiences during the measurement, participants filled out an OTP afterward which consisted of a blank page to freely report all personal subjective experiences occurring during the measurement. To place findings within the broader context of clinical research we included a measure of depressive symptomatology The overall goal of this study was to explore FC correlates of trait-like group differences in flourishing and SV with a focus on DMN activity and the mental processes of mind-wandering and rumination as potential explanatory variables.

## Materials and Methods

### Participants

Subjects were recruited using posters, flyers and the staff email distributor list of the University Hospital Tübingen. Among average healthy people the recruitment information explicitly asked for participants who felt a lot of energy or a high degree of well-being in their daily life. Additionally, data from 12 healthy subjects, who were part of the control group of a clinical intervention trial (NCT02375308) on depression with a similar experimental procedure, were used in this study. This study was carried out in accordance with the recommendations of ‘Ethical guidelines, Ethics Committee at the University Hospital and University of Tübingen’ with written informed consent from all subjects. All subjects gave written informed consent in accordance with the Declaration of Helsinki. The protocol was approved by the ‘Ethics Committee at the University Hospital and University of Tübingen.’ Only healthy subjects without acute or chronical coronary heart disease (e.g., hypertonia), diabetes or a diagnosed psychological or neurological disorder were included. Using an online questionnaire, 62 individuals were prescreened with regard to the exclusion criteria and their level of Fl and SV. Forty-three attended the laboratory session and provided data for the following analysis. Initially we planned on using “agreed” or “strongly agreed” on all items of one or both scales (≥48 for flourishing, ≥36 for SV) as classification criteria for “high” in the respective outcome (Hone et al., 2014). However, over the recruiting process it proved more difficult to find participants meeting this criterion for SV compared to flourishing (*n*_SV_ = 18 vs. *n*_Fl_ = 28). To keep group sizes equal and since we were interested in exploring extreme group effects without hypothesis on the effect of a clear cut-off value we used a median split approach (Farrington and Loeber, 2000) and assigned individuals with a score above the median (*m*_fl_ > 48; *m*_sv_ > 35) to the respective “high” group. 25 individuals were grouped as regSV and 18 as highSV, 23 as regFl and 20 as highFl. Twelve of the highSV subjects (i.e., 66%) also belonged to the highFl group. The group characteristics are displayed in Table 1. In the overall sample, 14% of the participants held a middle school degree, 83.7% a high-school diploma (German Abitur) and 2.3% a university degree. 69.8% were currently enrolled as students, 27.9% indicated to work full-time. 65% of the participants were female. Both high score subgroups did not differ from their low score counterparts with regard to age (for SV *t*_41_ = 0.99, *p* > 0.1; for Fl *t*_41_ = 0.95, *p* > 0.1), sex ratio (for SV χ^2^_1_ = 0.22, *p* > 0.1; for Fl χ^2^_1_ = 0, *p* > 0.1) and level of education (for SV χ^2^_2_ = 0.99, *p* > 0.1; for Fl χ^2^_2_ = 0.91 *p* > 0.1).

**Table 1:** Sample and subgroup characteristics.

	Normal-vital		High-vital			Normal-flourishing		High-flourishing		
	Mean	SD	Mean	SD	*t*/χ^2^	Mean	SD	Mean	SD	*t*/χ^2^
**Scale**	31.61	2.83	38.67	2.25		44.96	3.18	51.85	2.08	
**Age (years)**	27.5	6.55	30.72	12.46	*t*_(41)_ = 0.99 *p* > 0.1	31.00	11.36	27.96	9.56	*t*_(41)_ = 0.95 *p* > 0.1
**Sex (f/m)**	61.1%		68%		χ^2^_(1)_ = 0.22 *p* > 0.1	65%		65.2%	
***N*_subgroup_**	25		18			23		20	
***N*_highFl_**	8		12		***N*_highSV_**	6		12
***N*_normFl_**	17		6		***N*_normSV_**	17		8

**Overall sample (*n* = 43)**			**Mean**	**SD**	**Range**	**Kurtosis**	**Skewness**		**Cronbach α**	**Retest**

**Flourishing Scale**			48.16	4.402	38–56	−0.407	−0.402		0.782	*r*_(35)_ = 0.84
**Subjective Vitality Scale**			34.58	4.349	25–42	−0.487	−0.128		0.785	*r*_(36)_ = 0.87

*Subgroups were derived using a median split (>48 for high-flourishing, >35 for high-vital subjects). Participants with median value were assigned to the lower subgroup to balance group size.*

### fNIRS

**FIGURE 1:**
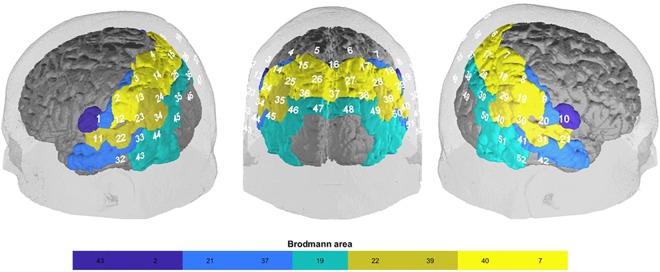
fNIRS-channel brain mapping (Brodmann area) based on a neuro-navigational measurement in an exemplary volunteer: somatosensory association cortex (BA 7; SAC; channel 4, 5, 6, 7, 15, 16, 17, 25, 26, 27, 28, 35, 36, 37), supramarginal gyrus (BA 40; SupG; channel 2, 3, 8, 9, 12, 13, 18, 19, 23, 30), angular gyrus (part of Wernicke’s area; BA 39; AngG; channel 14,24, 29, 34, 39, 40, 45, 50), superior temporal gyrus (BA 22; STG; channel 11, 21, 22, 31, 33, 41), visual area 3 (BA 19; V3; channel 38, 46, 47, 48, 49), fusiform gyrus (BA37; FusG; channel 43, 44, 51, 52), middle temporal gyrus (BA 21; MTG; channel 32, 42), primary somatosensory cortex (BA 2; PSC; channel 1, 20), subcentral area (BA43; SC; channel 10, 11).

Hemodynamic changes were measured via fNIRS, an optical imaging method using light in the near-infrared spectrum to measure concentration changes of oxygenated and deoxygenated hemoglobin. The penetration depth and therefore spatial measurement depth of fNIRS is approximately 2–3 cm (Haeussinger et al., 2014). Importantly, fNIRS has been shown to be a useful and reliable device to measure FC (Lu et al., 2010; Mesquita et al., 2010; Zhang et al., 2010; Deppermann et al., 2016; Rosenbaum et al., 2016). We used a continuous wave, multichannel NIRS system (ETG-4000 Optical Topography System; Hitachi Medical Co., Japan) with a temporal resolution of 10 Hz. The distance between channels was 3 cm. To measure parts of the DMN, we placed the probe set in the form of a rectangle over parietal areas covering the precuneus (Horn et al., 2014) with reference points Pz (Channel 16), T3 (Channel 43) and T4 (Channel 52), according to the 10-20 system (Jasper, 1958). The system consisted of 52 channels (Supplementary Figure 1). Channel positions with regard to Brodmann areas were located using a neuro-navigation system on a volunteer’s head (Figure 1).

### Procedure

The resting state measurement was part of a larger study (NCT02375308) on the cortical correlates of depression and well-being. Results regarding the depressive subsample are reported elsewhere (Rosenbaum et al., 2017). For the purpose of this study, data was assessed during a 7-min resting phase in which participants were asked to sit still with eyes closed, think of nothing in particular and let their thoughts flow. Since the participants had to complete other tasks as part of the overall study and RSFC has shown to be measurable reliably in short periods of time (Sakakibara et al., 2016; Zhao et al., 2016) we chose a 7-min resting-state measurement as a trade-off between data quality and economic demands.

#### Mind-Activity Measures

To assess thought processes and experiences during the measurement, directly after completion the subjects reported what they had done and experienced during measurement using (1) visual analog scales (VAS) and (2) a blank page for a written OTP (Rosenbaum et al., 2017). For the VAS, subjects were asked to approximately rate on a scale from 0 to 100% how much time they had spent on ten different activities (Rosenbaum et al., 2017). The scales of mind-wandering and rumination during the measurement were analyzed for this study. The free written OTP was screened and categorized by two independent raters to assess qualitative measures of the process during resting state according to qualitative methods: The forms were first analyzed and categories of experiential content were set and defined until saturation was reached. Second, the most common categories were used to categorize self-report forms by two independent raters. Also, the raters evaluated the emotional tone (positive, negative, mixed, neutral) and level of arousal (calm, aroused) of the thought protocol. For the final analysis, the ratings of the two independent raters for each OTP were discussed if deviating and integrated in a final single rating.

#### Trait Measures

Subjects were categorized based on their self-rating on scales of SV (Ryan and Frederick, 1997) and flourishing (Diener et al., 2010; Esch et al., 2012). Both scales where phrased to be answered with regard to life in general using a Likert scale format (strongly disagree – strongly agree). The SV scale consists of six items to assess a person’s self-perceived level of energy (e.g., Item 1: “I feel alive and vital”; Item 3: “I have energy and spirit”) and alertness (e.g., Item 5: “I nearly always feel alert and awake”) in daily life. Diener et al. (2010) proposed eight items to determine a person’s level of flourishing. The scale covers aspects of self-perceived meaning and purpose (Item 1: “I lead a purposeful and meaningful life”), engagement (Item 3: “I am engaged and interested in my daily activities”), competence (Item 5: “I am competent and capable in the activities that are important to me”), self-esteem (Item 6: “I am a good person and live a good life”), optimism (Item 7: “I am optimistic about my future”) and quality in relationships (Items 2, 4, 8 e.g., “My social relationships are supportive and rewarding”). Trait rumination was assessed using the subscale rumination of the ruminative response scale (RRS; Nolen-Hoeksema and Morrow, 1991). To control for associations with depressive symptomatology we included the depression module of the Patient Health Questionnaire (PHQ-9; Kroenke et al., 2001).

### Data Preprocessing

The data was processed and analyzed using MATLAB R2017b (MathWorks Inc., Natick, MA, United States, **RRID**:SCR_001622). After preprocessing, the MATLAB *NBS toolbox* (Zalesky et al., 2010; **RRID**:SCR_002454), *Wavelab850 toolbox*^1^ and *BrainNetViewer toolbox*^2^ (Xia et al., 2013; **RRID**:SCR_009446) were used for analyzing and plotting results. Furthermore, SPSS (Version 24; **RRID:SCR_002865**) was used for data analysis. fNIRS data preprocessing included: bandpass filtering (0.1–0.01 Hz, FC differences were expected in this spectrum) to minimize high- and low-frequency noise, movement artifact reduction by correlation-based signal improvement (Cui et al., 2010; Brigadoi et al., 2014), as well as component-based removal of bite artifacts (ICA). For the resting state subjects were instructed to keep their heads as still as possible and refrain from clenching their teeth. Afterward, all signals were visually inspected which revealed noisy channels after the described preprocessing in seven subjects. In these cases, channels were interpolated from surrounding channels. Three (one subject) or one channel (six subjects) had to be interpolated. Since FC can be significantly influenced by global signal changes, e.g., low frequency blood pressure oscillations (Mesquita et al., 2010), a global signal reduction was performed with a spatial Gaussian Kernel filter (Zhang et al., 2016) with a standard deviation of σ = 50. No short distance channels were used. After preprocessing, FC-coefficients were computed for each participant using pairwise correlation between all channel’s signal time courses. The values were then transformed via Fishers r-to-z-transformation (Silver and Dunlap, 1987).

### Network-Based Statistics (NBS)

Subsequent FC-differences between the flourishing and the SV subgroups were investigated with NBS (Zalesky et al., 2010). NBS is a statistical method that uses massive univariate testing of a contrast on connectivity matrices, and clusters connections that exceed a significance threshold using a breadth first search. The significance of the extracted cluster is then tested using permutation tests. The resulting *p*-values represent the likelihood to attain a cluster, similar or larger in the number of connected edges under the assumption of random group assignment of the individual scores in the sample at hand. Settings for NBS were set as follows: statistical threshold for massive univariate testing was set at *t* = 3.1, significance level for permutation tests α = 0.05, permutations = 5000, component size = “extent.” We estimated confidence intervals for the computed *p*-values of the permutation tests parametrically following Zalesky et al. (2010).

### Analysis Procedure

The following analysis was performed on the data: After the computation of FC measures, NBS were used to identify network-differences in FC between the highFl (score > 48) and the regFl group as well as between the highSV (score > 35) and the regSV group. Group differences were calculated using independent *t*-tests and chi-squared tests for the VAS, trait rumination, the OTP and depressive symptomatology. Whenever stated, significance levels for these tests were adjusted for multiple comparisons using the Bonferroni correction method. Significant group differences in mind-wandering, rumination and depressive symptomatology were used as covariates in the NBS models to test their role as explanatory variables for differences in FC patterns. In case of a significant influence of the covariate on the NBS its influence was further explored via the examination of correlations between the covariate and the significant network connections. To further explore the relation between SV and flourishing, we calculated NBS for one variable using the other as covariate and calculated correlations between the covariate and the significant network connections of the hub nodes in each network. Eventually, hub nodes (≥3 edges) of the significant networks were used as seed regions and the group comparisons in network connectivity strength were plotted. The fNIRS raw data as well as the respective code script and SPSS file are available under https://doi.org/10.17026/dans-zym-vewk.

## Results

### Flourishing

**FIGURE 2:**
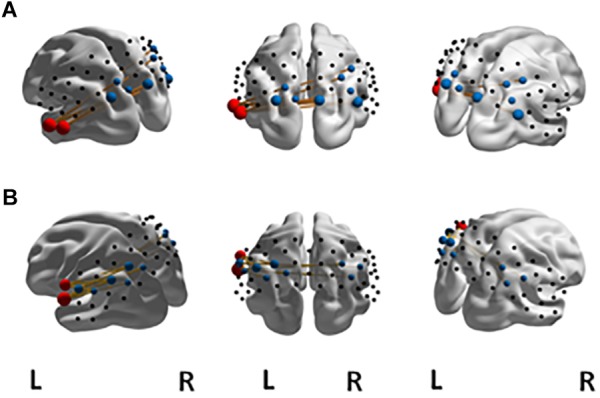
Significant NBS network at *t* = 3.1 for FC in the **(A)** high-flourishing group compared to the regular-flourishing group with hub nodes (red) at left MTG and left FusG, other network nodes (blue) and edges (yellow). The significant NBS network at *t* = 3.1 for FC in the high-vital group compared to the regular-vital group with hub nodes (red) at left sub central area and left primary somatosensory cortex is displayed in **(B)**.

**FIGURE 3:**
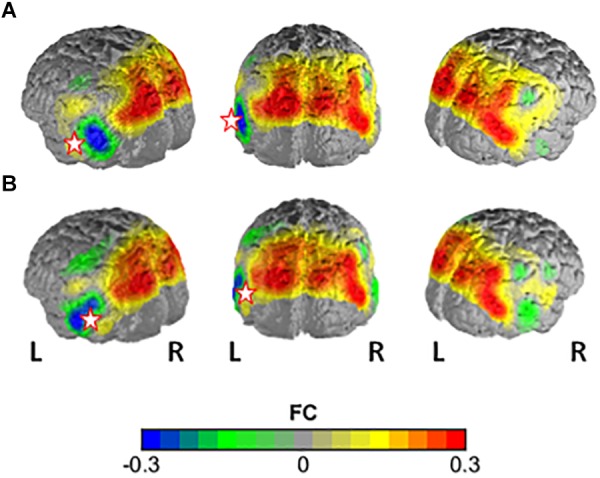
Difference in FC between the high-flourishing and regular-flourishing group with **(A)** channel 32 (lMTG) and **(B)** channel 43 (lFusG) as seed regions. Warm colors indicate higher FC with seed in high-flourishers vs. low-flourishers.

The NBS yielded a single more strongly connected network for the highFl group, comprising 11 functional connections at threshold *t* = 3.1 (*p* = 0.036 ± 0.0053). The derived network consisted of 10 nodes with 11 edges (Table 2). Nodes were classified as hub nodes if they had more than **three edges**. The network centered around two hub nodes in the left middle temporal (MTG) and the left FusG, spreading onto bilateral parietal areas of the DMN (Figure 2A), bilateral parts of the SAC and visual area (V3). The right angular (AnG) and SuG were part of the network in the right hemisphere. Further analysis revealed that flourishing correlated significantly positively with all except two connections in the network (*p* < 0.1). All correlations are displayed in Table 3. The differences in FC between regFl and highFl participants are displayed in Figure 3 using the two hub nodes left MTG (A) and left FusG (B) as seed channels.

**Table 2:** Degrees of the significant network differences between high-flourishing and regular-flourishing subjects (*t* = 3.1) and high-and regular-vital subjects (*t* = 3.1).

Channel	Region	Flourishing (*t* = 3.1) Degree	Sub. Vitality (*t* = 3.1) Degree
1	PSC (left)		**3**
11	SC/STG (left)		**5**
12	SupG (left)		2
18	SupG (right)	1	
23	SupG (left)		1
24	AngG (left)		2
25	SAC (left)		2
28	SAC (right)	1	1
29	AngG (right)		1
32	MTG (left)	**6**	
35	SAC (left)		1
36	SAC (left)	1	1
39	AngG (right)	1	1
43	FusG (left)	**5**	
46	V3 (left)	2	
47	V3 (left)	1	
48	V3 (right)	2	
50	AngG (right)	2	
Nodes		10	11
Edges		11	10
*p*-value		0.036 ± 0.0053	*p* = 0.046 ± 0.0059

*Only channels of the significant networks are presented. PSC, primary somatosensory cortex; SC, subcentral area/secondary somatosensory cortex; SupG, supramarginal gyrus; AnG, angular gyrus; SAC, somatosensory association cortex; MTG, middle temporal gyrus; FusG, fusiform gyrus; V3, visual area. Hub nodes marked in bold.*

**Table 3:** *P*-Values of the significantly stronger connected network channels in the flourishing network and correlations with flourishing, subjective vitality, mind-wandering, trait rumination, and depression.

Hub nodes (seed)		*t* = 3.1	Flourishing		Subjective vitality		Mind-wandering		Trait rumination		Depression	
		*p*-value	*r*	*p*	*r*	*p*	*r*	*p*	*r*	*p*	*r*	*p*
**Flourishing network**												
lMTG (Ch 32)	rSupG (Ch 18)	0.002	0.39^∗^	0.01	0.13	0.387	0.30	0.05	−0.20	0.204	−0.31^∗^	0.041
	lSAC (Ch 36)	0.001	0.26	0.095	0.21	0.172	0.29	0.058	−0.28	0.071	−0.36^∗^	0.018
	lV3 (Ch 46)	0.001	0.26	0.096	0.16	0.303	0.35^∗^	0.023	−0.33^∗^	0.029	−0.20	0.203
	lV3 (Ch 47)	0.002	0.22	0.158	0.18	0.252	0.39^∗^	0.009	−0.30	0.05	−0.27	0.077
	rV3 (Ch 48)	0.002	0.29	0.062	0.18	0.241	0.44^∗^	0.003	−0.32^∗^	0.036	−0.31^∗^	0.04
	rAngG (Ch 50)	0.003	0.21	0.174	0.20	0.195	0.26	0.096	−0.26	0.089	−0.21	0.166
												
lFusG (Ch 43)	rSAC (Ch 28)	0.003	0.36^∗^	0.018	0.17	0.284	0.22	0.146	−0.35^∗^	0.021	−0.38^∗^	0.012
	rAngG (Ch 39)	0.002	0.31^∗^	0.04	0.12	0.451	0.19	0.231	−0.28	0.065	−0.34^∗^	0.028
	lV3 (Ch 46)	0.002	0.29	0.06	0.17	0.278	0.30	0.051	−0.42^∗^	0.005	−0.30^∗^	0.047
	rV3 (Ch 48)	0.001	0.30	0.053	0.15	0.34	0.43^∗^	0.004	−0.37^∗^	0.014	−0.35^∗^	0.021
	rAngG (Ch 50)	0.003	0.26	0.086	0.26	0.086	0.16	0.305	−0.33^∗^	0.032	−0.27	0.074

*All variables were used as covariates in a follow-up NBS analysis of flourishing. ^∗^p < 0.05; the p-value corrected for multiple comparison was *p* < 0.0045.*

### Subjective Vitality

**FIGURE 4:**
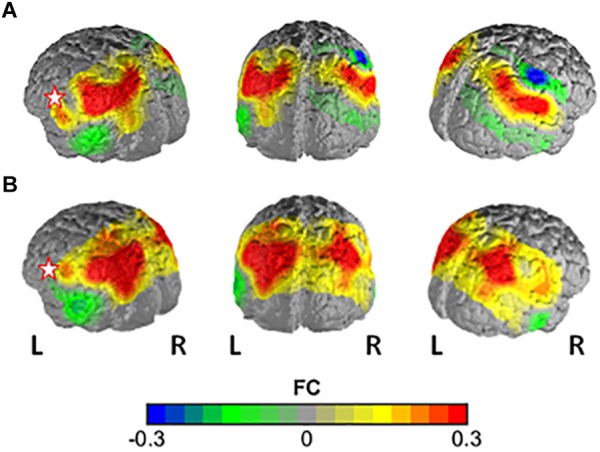
Difference in FC between the high-vital and the regular-vital group with **(A)** channel 1 (PSC) and **(B)** channel 11 (lSC) as seed regions. Warm colors indicate higher FC with seed in high-vital vs. regular-vital participants.

In the comparison of the highSV and the regSV group, the NBS analysis yielded a significantly more strongly connected network for the highSV group comprising 10 functional connections at threshold *t* = 3.1 (*p* = 0.046 ± 0.0059). The network consisted of 11 nodes and 10 edges (Table 2). The major hub node was located in an overlapping area of the left subcentral area (SC) and superior temporal gyrus, connecting to nodes in the bilateral SAC and the bilateral AnG. The second most connected node within the PSC stretched to left SupG, left AnG and left SAC (Figure 2B). The network did not reach significance (*p* < 0.05) at any other threshold, however, different thresholds returned *p*-values close to the level of significance (*t* = 2.8, *p* = 0.0721, *t* = 3.0, *p* = 0.0546; *t* = 3.2, *p* = 0.0618; *t* = 3.3, *p* = 0.0552). In depth analysis revealed that SV correlated positively with all except one connection in the network (*p* < 0.05; Table 4). The differences in FC between regSV and highSV participants are displayed in Figure 4 using the two hub nodes left PSC and left SC as seed channels.

**Table 4:** *P*-Values of the significantly stronger connected network channels in the subjective vitality network and correlations with flourishing, subjective vitality, mind-wandering, trait rumination, and depression.

Hub nodes (seed)		*t* = 3.1	Flourishing		Subjective vitality		Mind-wandering		Trait rumination		Depression	
		*p*-value	*r*	*p*	*r*	*p*	*r*	*p*	*R*	*p*	*r*	*p*
**Subjective vitality network**												
lSC/STG (Ch 11)	lAngG (Ch24)	0.002	0.17	0.261	0.33^∗^	0.033	−0.03	0.856	0.02	0.916	−0.16	0.30
	rSAC (Ch 28)	0.001	0.24	0.124	0.33^∗^	0.031	−0.02	0.916	0.06	0.682	−0.02	0.873
	lSAC (Ch35)	0.003	0.24	0.120	0.36^∗^	0.018	0.13	0.387	0.05	0.77	−0.03	0.826
	lSAC (Ch36)	0.001	0.14	0.366	0.41^∗^	0.006	−0.06	0.678	0.17	0.277	−0.04	0.811
	rAngG (Ch39)	0.003	0.20	0.199	0.33^∗^	0.03	−0.095	0.546	0.11	0.497	0.05	0.735
												
lPSC (Ch 1)	lSupG (Ch23)	0.001	0.20	0.19	0.35^∗^	0.023	−0.17	0.277	−0.10	0.52	−0.11	0.491
	lAngG (Ch24)	<0.001	0.15	0.345	0.34^∗^	0.026	−0.21	0.17	−0.09	0.553	−0.08	0.63
	lSAC (Ch25)	0.002	0.18	0.258	0.40^∗^	0.008	−0.18	0.234	−0.05	0.743	−0.03	0.848
												
SupG (Ch 12)	lSAC (Ch 25)	0.003	0.08	0.623	0.34^∗^	0.027	−0.25	0.102	0.06	0.719	0.08	0.588
	rAngG (Ch 29)	0.006	0.24	0.121	0.18	0.241	−0.21	0.183	−0.17	0.261	−0.05	0.741

*All variables were used as covariates in a follow-up NBS analysis of flourishing. ^∗^p < 0.05; the p-value corrected for multiple comparison was p < 0.0045.*

### Covariate Networks and the Relation Between Flourishing and Subjective Vitality

Subjective vitality was positively correlated with flourishing (*r* = 0.63, *p* < 0.001). When using SV as a covariate in the NBS procedure for flourishing, the significant network difference between the flourishing groups dissolved. However, in depth analysis of the correlations between SV and the network connections in the flourishing network yielded only one marginally significant correlation with the connection between lFusG and lAngG (*r* = 0.26, *p* = 0.086). All correlations are displayed in Table 3. Entering flourishing as a covariate into the NBS analysis of SV, yielded a significant network comprising 11 nodes and 11 edges at *t* = 3.1 (*p* = 0.044 ± 0.0058). In contrast to the original vitality network, this network remained significant at different thresholds. At *t* = 2.3 a significant network resulted comprising 29 nodes and 67 edges (*p* = 0.040 ± 0.0055). At *t* = 2.7 the network decreased to 21 nodes and 35 edges (*p* = 0.028 ± 0.0047). Flourishing showed no significant correlations with any of the connections of the SV network (all *p* > 0.1; Table 4).

### Rumination, Mind-Wandering, the OTP and Depressive Symptomatology

HighFl participants reported significantly more mind-wandering than regFl participants (*t*_32.2_ = 4.34, *p* < 0.001 *d* = 1.30), significantly less trait rumination (*t*_41_ = 3.20, *p* = 0.003, *d* = 0.99) and significantly less depressive symptomatology (*t*_34.5_ = 2.52, *p* = 0.017, *d* = 0.75); no significant difference between groups was found for state-rumination. The highSV and the regSV group did not differ significantly on mind-wandering, depressive symptomatology, state or trait rumination. Both high-score groups did not differ from their regular counter parts with respect to any category of the OTP. The categories derived for the OTP and content classification percentages are presented in the Supplementary Table 1. The only exception was the extent of thinking about the measurement for the flourishing groups (25% of regFl vs. 5% of highFl; χ^2^_1_ = 7.26, *p* < 0.004 corrected for multiple comparison, OR = 8.25). Also, no group differences were found for emotional tone and experienced arousal.

### Rumination, Mind-Wandering, and Depressive Symptomatology as Covariates in the NBS

Because trait rumination, mind-wandering and depressive symptomatology differed significantly between the highFl and the regFl group we conducted further analysis and used all three variables as covariates in a repeated NBS analysis of flourishing. Using the degree of mind-wandering in the NBS for the flourishing groups as a covariate rendered the network insignificant. A closer examination of the relation between mind-wandering and FC within the flourishing network, when using the hub node in the left MTG as a seed region, revealed significantly positive correlations for six network connections (*p* < 0.1). However, only the relation with the right visual area remained significant after correction for multiple comparisons (*r*_43_ = 0.44, *p* < 0.0045). Using the second hub node within left FusG as a seed yielded positive correlations for the connection with the left visual area (*r* = 0.30, *p* = 0.051) and right visual area (*r* = 0.43, *p* = 0.004). The latter remained significant after correction for multiple comparisons (*r* = 0.43, *p* < 0.0045). All correlations are displayed in Table 3.

When trait rumination was entered as a covariate, also no significant network resulted as a difference between groups. Further analysis revealed FC within the flourishing network to be negatively correlated with trait rumination. When using the left MTG as a seed, significant negative correlations were found for FC with left (*r* = −0.33, *p* = 0.029) and right visual area (*r* = −0.32, *p* = 0.036). The associations with left SAC (*r* = −0.28, *p* = 0.071) and right angular gyrus (*r* = −0.26, *p* = 0.089) pointed toward a significant correlation. No correlation survived correction for multiple comparisons (*p* > 0.0045). When taking the left FusG as a seed region correlations between trait rumination and FC with bilateral visual area (−0.42 < *r* < −0.37, 0.005 < *p* < 0.01), right AnG (*r* = −0.33, *p* = 0.032) and rSAC (*r* = −0.35, *p* = 0.021) turned out significant. No correlation remained significant after correction for multiple comparisons. All correlations are displayed in Table 3.

Using depressive symptomatology as a covariate in the NBS yielded no significant connectivity network difference between the highFl and the regFl group. Further correlational analysis revealed negative correlations between depression and the connectivity strength between lMTG and rSupG (*r* = −0.31, *p* = 0.041), lSAC (*r* = −0.36, *p* = 0.018) and rV3 (*r* = −0.31, *p* = 0.041). Furthermore, depressive symptomatology correlated negatively with the connectivity strength between lFusG and rSAC (*r* = −0.38, *p* = 0.012), rAngG (*r* = −0.34, *p* = 0.028), lV3 (*r* = −0.30, *p* = 0.047) and rV3 (*r* = −0.35, *p* = 0.021). No correlation survived correction for multiple comparison (*p* < 0.0045).

In case of the NBS for subject vitality, mind-wandering as a covariate led to a decrease of the original network comprising seven nodes with six edges at *t* = 3.2 (*p* = 0.047 ± 0.0060). The original network remained stable when trait rumination was added as a covariate in the NBS (*p* = 0.049 ± 0.0061). Adding symptoms of depression as covariate lead to no significant group differences in connectivity strength at threshold of *t* = 3.1. However, a marginally significant difference resulted at threshold *t* = 2.9 (*p* = 0.0902 ± 0.0081). All correlations of the covariates with the SV network are displayed in Table 4.

## Discussion

The goal of this study was to investigate associations of cortical FC at rest with two widely used indicators of well-being – flourishing and SV. For people high in flourishing, we found significantly increased FC within a network comprising parts of the DMN (right angular gyrus, right SuG, left MTG), bilateral somatosensory and visual cortex and left FusG. For highSV participants, we found a network of significantly increased FC related to the DMN (bilateral angular gyrus, left SuG, left superior temporal gyrus) and nodes in bilateral somatosensory, left primary and secondary somatosensory cortex. The inclusion of either mind-wandering, trait rumination or depression as covariate in the NBS nullified the difference in FC between the flourishing groups. In comparison, the vitality network remained, when including mind-wandering or trait rumination as a covariate in the NBS. Depressive symptomatology as covariate led to a marginally significant difference at a lower threshold.

### DMN

Our results add to prior findings of the association between changes in DMN FC and trait indicators of well-being (Luo et al., 2015, 2017). However, depending on the measure of well-being and the specific area of the DMN, the authors reported heterogeneous findings regarding the increase and decrease of FC. In our study, which was limited to parietal and temporal cortex areas, we observed increased FC for areas that included the bilateral inferior parietal lobe (AnG/SuG) and left lateral temporal areas.

### Flourishing

The network of increased FC within the highFl group centered around two hub nodes in the left MTG and the left FusG. As part of the DMN, the MTG has been associated with the provision of memory content in the process of spontaneous thought generation (Smallwood et al., 2016), but also social information processing in general (Alcalá-López et al., 2017). Behavioral research shows that the DMN related activity of mind-wandering is crucial for the navigation of the social world (Poerio and Smallwood, 2016) and in turn, social day-dreaming is being associated with increased feelings of love, connectedness and happiness (Poerio et al., 2015). We believe this is a potential dynamic behind the results in this study as a major factor in the selection of individuals as highFl was the reported quality of their social relationships. HighFl participants showed higher ratings of social commitment for others and perceived support and respect in their relationships (three out of seven items). Our findings of increased FC in social- and DMN-related brain areas in highFl individuals and the role of mind-wandering indicate a link between three different lines of research: The ”social brain” (Tan et al., 2014; Alcalá-López et al., 2017), DMN-related spontaneous thought activity (Smallwood et al., 2016) and the importance of social factors for well-being (Diener and Seligman, 2002; Kafetsios and Sideridis, 2006; Sánchez-Álvarez et al., 2016). The FusG as second hub node in the flourishing network and its’ role in face recognition (Kanwisher and Yovel, 2006) with relevance for social cognition and emotional intelligence (Takeuchi et al., 2011, 2013) lent further support to this hypothesis. The co-appearance of left MTG and FusG in our findings is also in line with a PET study by Volkow et al. (2011) which found positive emotionality, a construct composed of well-being, achievement/motivation, social potency and social closeness, to be positively associated with glucose metabolism in the left MTG and FusG. Overall, our results for flourishing and brain activity are consistent with studies that suggest a link between the processing of social cues, DMN activity and increased levels of well-being. At the same time they provide support for a mechanism underlying the prominent broaden-and built theory of positive emotion (Fredrickson, 2001). Multiple behavioral studies have supported the claim that positive emotions broaden our scope of attention and foster a state of learning (Fredrickson, 2013); our findings indicate an extension to the neurophysiological level via the link of differences in DMN related FC, mind-wandering and trait levels of flourishing.

### Subjective Vitality

The two hub nodes in the vitality network were located in the left primary and secondary somatosensory cortex (Eickhoff et al., 2006) overlapping with posterior left superior temporal gyrus. Individuals high in trait SV report prolonged feelings of increased aliveness and energy, which is in line with findings of a connection between PSC and arousal and attention related areas of the brain (Gobbelé et al., 2000; Jang et al., 2014). A higher level of perceived energy can also be achieved via anodal transcranial direct current stimulation (tDCS) of the bilateral PSC (Tecchio et al., 2014). The posterior superior temporal gyrus has been linked to DMN activity (Wang et al., 2015) whereas secondary somatosensory cortex has been associated with the unconscious representation of feelings and peripheral physiological activity (Anders et al., 2004a,b). The frequent experience of elated, positive states associated with physiological arousal in highSV individuals (Ryan and Bernstein, 2004) is in line with these findings. On a higher level, PSC and somato-associative cortex play a role in the feeling of ownership and identification with one’s own body (Aspell et al., 2012; Blanke, 2012). This form of body-connection, in turn, is positively associated with physical activity (Babic et al., 2014). Results from this study sample (reported elsewhere) suggest that the highSV group (*M* = 8.34, *SD* = 4.23) spends significantly more hours on physical activity per week (*t*_32_ = 3.54, *p* = 0.001) than the regSV group (*M* = 3.72, *SD* = 3.37). However, this difference does not exist for highFl and regFl individuals (*t*_32_ = 0.28, *p* = 0.781). The assumption that highSV individuals may be more prone to body-related self-processing relates to the DMN literature as Treserras et al. (2009) found that sensorimotor networks become coupled with DMN networks when preparing for movement or activity; a state which, according to the authors, can last over longer periods of time and may be one explanation for the findings regarding SV in this study. In contrast to flourishing, entering mind-wandering as covariate only decreased the size of the FC network difference between highSV and regSV participants. This is consistent with the fact that part of the vitality network shows DMN overlap whereas the major hub nodes in the primary and secondary somatosensory cortex are not considered part of the DMN.

### Flourishing and Subjective Vitality

Despite the conceptual overlap of SV and flourishing, the body as a stage for subjective experience (Damasio et al., 2000) may be more prominent in highSV individuals. HighFl individuals on the other hand, are a selection of people with strong positive cognitive evaluations of life (e.g., the self, social relationships, the future). In their joint NBS analysis, flourishing as a covariate stabilized the vitality network, whereas SV as a covariate dissolved the flourishing network. We speculate that adding cognitive DMN related components of flourishing on top of a body-related vitality core component increases the network, whereas taking the body-related core away removes variance of a more fundamental component that is nevertheless central to flourishing. Adding to the argument of a different role of cognition in the two well-being measures is the finding that habitual (rumination) and spontaneous (mind-wandering) thought processes explained main shares of variance in FC between the flourishing but not the SV groups. State rumination did not significantly differ between groups and was not used further as covariate in the analysis. On the one hand, a mere resting state procedure may not be an adequate measure to assess healthy people’s spontaneous tendency to ruminate (Rosenbaum et al., 2017); on the other hand, the experience of spontaneously flowing thoughts may just be of higher discriminative power regarding the extent of well-being in non-clinical samples. Overall, we speculate the high correlation between flourishing and SV and their distinct relation to states of mind indicate essential overlap between the two constructs with potential differences in higher order brain processes (Kringelbach and Berridge, 2017).

### Flourishing, Subjective Vitality, and Symptoms of Depression

Of further interest is the fact that in this study we found depressive symptomatology to be negatively correlated with flourishing on a behavioral and neurophysiological level. The findings support the notion of an anti-relation between flourishing and mental illness (Huppert and So, 2013). For SV, the inclusion of depressive symptoms in the NBS analysis weakened the group differences on a neurophysiological level and no relation was found on a behavioral level. Depression showed no significant correlation with any of the significant network relations in the SV network which speaks to the fact that the NBS result may be more of a power problem. If so, the findings are in line with research that shows well-being/positive valence as a distinct phenomenon which goes beyond the mere opposite of malicious states (Keyes, 2002; Cuthbert and Insel, 2013). Among a more differentiated diagnostic, these findings may be relevant for the creation and effect of interventions where improvement and prevention of states of illness may demand different foci. Further research on the neurophysiology of positive states and traits could help to illuminate what is needed for each segment.

### Limitations

One major limitation of this study was the restriction on parietal cortical areas of the DMN. Due to its usability and robustness against artifacts, fNIRS is a promising method to study brain activity and spontaneous thought processes in naturalistic contexts. However, this comes at the cost of limited insight into the activity of deeper-lying brain structures and whole brain activity. In case of this study, no conclusions can be drawn about medial and frontal subcomponents of the DMN. Secondly, we used NBS to identify significant differences in brain activation between groups. This approach allows for an interpretation on a network-level; conclusions on the role of single nodes have to be taken with care. Differences in DMN activity during rest have been related to group differences as well as various types of self-generated thought. However, due to the lack of experimental control during the resting state, the interpretability of on-going mind and brain processes within the participant is limited. We tried to control for this via the collection of OTP data from each participant after the measurement. However, we did not find any significant difference with regard to the content and emotional tone reported by the participants in the different groups. Meyer et al. (2015) reported changes in brain activity following imagined relieve of physical pain which did not display in the self-report of participants following their measurement. This adds to our findings, as the role of subconscious processes and lack of information about the on-going experience of the participant are two major limitations that need to be considered in the interpretation of our results. A further constraint in this study was the limitation of statistical power to detect medium and small effect sizes due to the modest sample size. In the case of the NBS for SV and the in-depth analysis of covariates a number of results were significant only at the level of α = 0.10 and often did not survive correction for multiple comparison. One major strength of NBS is the increase in statistical power (Zalesky et al., 2010) that comes at the cost of limited interpretational power of single network connections. We therefore believe, the results of this study should be considered a starting ground that needs to be tested and extended in future studies.

## Conclusion

In the well-being literature, conceptual distinctions have been made between eudaimonic and hedonic components of well-being (Ryan and Deci, 2001; Peterson et al., 2005). Others have separated cognitive from affective or global from specific aspects of subjective well-being (Diener, 1984; Diener et al., 1999). Flourishing has evolved as a complex construct in response to the diversity in symptomatology of psychological disorders. SV on the other hand specifically addresses the link between the subjective experience and organismic processes rooted in the human body. Hence, both constructs are distinct from other constructs used in the existing well-being literature. A neurophysiological framework to integrate the different concepts is still lacking. Our results add to the existing literature by showing distinct cortical FC correlates of flourishing and SV in the brain at rest. This may serve the purpose of further unraveling the neurophysiological correlates of the good life.

## Data Availability Statement

The fNIRS raw data as well as the respective code script and SPSS file can be found in the EASY DANS repository under https://doi.org/10.17026/dans-zym-vewk.

## Author Contributions

FG did the primary drafting, interpretation of the data and data analysis. DR contributed to the analysis of the data. AH contributed to the drafting of the experimental design and acquisition of the data. TR, MH, AF, and A-CE contributed to the design and the acquisition of the work and revised it critically for important intellectual content.

## Conflict of Interest Statement

The authors declare that the research was conducted in the absence of any commercial or financial relationships that could be construed as a potential conflict of interest.

## Footnotes

1 http://statweb.stanford.edu/~wavelab/

2 http://www.nitrc.org/projects/bnv

## Abbreviations

Ang: angular gyrus
DMN: default mode network
FC: functional connectivity
Fl: flourishing
fNIRS: functional near-infrared spectroscopy
FusG: fusiform gyrus
highFl: high-flourishing
highSV: high-vital
IPL/AG: inferior parietal lobe/angular gyrus
MPFC: medial prefrontal cortex
MTC: medial temporal lobe
MTG: middle temporal gyrus
NBS: network-based statistics
OTP: open-thought protocol
PCC/Rsp: posterior cingulate/retrosplenial cortex
PSC: primary somatosensory cortex
regFl: regular-flourishing
regSV: regular-vital
RRS: ruminative response scale
RSFC: resting state functional connectivity
SAC: somatosensory association cortex
SC: subcentral area/secondary somatosensory cortex
SuG: supramarginal gyrus
SV: subjective vitality
V3: visual area
VAS: visual analog scale
