# Supplementary material for: The Positive Brain – Resting State Functional Connectivity in Highly Vital and Flourishing Individuals
Source: Front Hum Neurosci. 2019 Jan 14;12:540. doi: 10.3389/fnhum.2018.00540 (PMC6339902; doi:10.3389/fnhum.2018.00540)
Supplement: Supplementary file 1 [file Data_Sheet_1.docx]

Supplementary Material

The Positive Brain – Resting State Functional Connectivity in Highly Vital and Flourishing Individuals

Florens Goldbeck^1*^, Alina Haipt^1^, David Rosenbaum^1^, Time Rohe^1^, Andreas J. Fallgatter^1,2^, Martin Hautzinger^3^, Ann-Christine Ehlis^1,2^

*** Correspondence:** Florens Goldbeck: florens.goldbeck@uni-tuebingen.de

# Supplementary Table 1. Categories of the OTP, example and % occurrence in overall sample

|  | Category subjective experience | Example | % |
| --- | --- | --- | --- |
| 1 | Mind-wandering: The subject expressed to be in a relaxed mood and let his mind flow in an unconstrained way without any focus on a particular subject | “I relaxed and let my mind flow.” | 25 |
| 2 | Rumination: The subject expressed a repetitive stressful style of thinking about an unfinished concern that leads to the urge of suppressing the inner experience. | “I thought about a stressful meeting I had at work, which made me nervous, so I tried to distract myself from that memory.” | 5 |
| 3 | Focus on body sensations: The subject expressed an attentional focus on their body/breath | “I focused on my breathing.” | 35 |
| 4 | Mindfulness/Relaxation training: The subject expressed to be in a mindful state (detachment from cognition, concentration on breathing with detached mind) or 3 to perform some kind of relaxation technique (e.g. progressive muscle | “I focused on my breathing and watched my mind in a detached way.” | 12 |
| 5 | Suppression: The subject expressed withdrawal from or suppression of unpleasant inner experiences | "I felt the weight (of the NIRS cab) on my head and tried to think about something else" | 12 |
| 6 | Boredom: The subject expressed that the resting-state was boring | "I felt bored" | 5 |
| 7 | Making future plans: The subject expressed thoughts about things they will do | “I thought about what I would eat for dinner and decided to eat pizza.” | 60 |
| 8 | Thinking about the measurement: The subject expressed thoughts about the given instructions or how their data might look like | "I wondered what he (the experimenter) would see" | 30 |
| 9 | Fight against fatigue: The subject expressed feeling sleepy or trying not to fall asleep | "I felt tired | 32 |
| 10 | Thinking about the duration of the measurement | "I was wondering how much time had already passed" | 21 |
| 11 | Listening to external noise: The person reported focusing on external sounds | "I heard the noise of the NIRS machine" | 5 |

|  | Overall emotional tone of content: |  |  |
| --- | --- | --- | --- |
|  | Positive | "I was thinking about my last surf vacation and how good it felt to be in the water." | 47 |
|  | Negative | "I felt upset about my PhD thesis and kept thinking how much I still needed to write" | 18 |
|  | Neutral | "Then I did some mental calculus and repeated the movements I had learned yesterday | 17 |
|  | Mixed | "The lecture was very boring but in the evening I met my friends which was nice" | 13 |
|  | Aroused | "I was feeling excited" | 11 |
|  | Calm | "I felt quiet and didn't think much" | 48 |

**Supplementary Fig 1**.


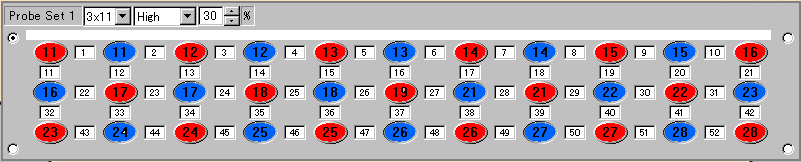


The 52-channel probe set was located using Pz, T3 and T4 of the 10-20 system as reference points.
